# The impact of rapport on intelligence yield: police source handler telephone interactions with covert human intelligence sources

**DOI:** 10.1080/13218719.2020.1784807

**Published:** 2020-07-30

**Authors:** Jordan Nunan, Ian Stanier, Rebecca Milne, Andrea Shawyer, Dave Walsh, Brandon May

**Affiliations:** a Institute of Criminal Justice Studies, University of Portsmouth, Portsmouth, UK; b School of Justice Studies, Liverpool John Moores University, Liverpool, UK; c School of Law, De Montfort University, Leicester, UK

**Keywords:** Covert Human Intelligence Source, human intelligence, HUMINT, intelligence, interviewing, rapport, source handler

## Abstract

Covert Human Intelligence Sources (CHIS) provide unique access to criminals and organised crime groups, and their collection of intelligence is vital to understanding England and Wales’ threat picture. Rapport is essential to the establishment and maintenance of effective professional relationships between source handlers and their CHIS. Thus, rapport-based interviewing is a fundamental factor to maximising intelligence yield. The present research gained unprecedented access to 105 real-life audio recorded telephone interactions between England and Wales police source handlers and CHIS. This research quantified both the rapport component behaviours (e.g., attention, positivity, and coordination) displayed by the source handler and the intelligence yielded from the CHIS, in order to investigate the frequencies of these rapport components and their relationship to intelligence yield. Overall rapport, attention and coordination significantly correlated with intelligence yield, while positivity did not. Attention was the most frequently used component of rapport, followed by positivity, and then coordination.

## Introduction

Everybody wants to talk. My job is to become the person he wants to talk to. (McCauley, 2007, p. 399)

The collection of detailed, timely and reliable information plays a vital role for law enforcement agencies in bringing criminals to justice (Stanier & Nunan, 2018). The received *actionable* information, formally recognised as intelligence (Grieve, 2004), informs critical law enforcement decision-making concerning intelligence-led operations and investigative enquiries. One intelligence collection tactic is the official use of informants, deployed to provide insider access to criminal activity and targets of interest. Within England and Wales, an informant is legally defined as a Covert Human Intelligence Source (CHIS) under Section 26(8) Part II of the Regulation of Investigatory Powers Act 2000, if they establish or maintain a relationship with another person to obtain information covertly; give access to information on another person; or disclose information covertly that they have obtained using the relationship or they have obtained because the relationship exists.

In England and Wales, CHIS report their intelligence to law enforcement officers, known as source handlers within policing. Source handlers are dedicated intelligence officers who are legally accountable for the security and welfare of their CHIS. As such, source handlers operate out of local (i.e. Basic Command Unit/Force Units) regional (i.e. Regional Organised Crime Units or Counter-Terrorism Units) or national (i.e. National Crime Agency) Dedicated Source Units. Up to 80% of the overt work undertaken by law enforcement personnel comprises the elicitation of information through *purposive conversations* (Newberry, 1997), although with source handlers, the daily interaction with CHIS is on a covert basis. CHIS should be considered a cost-effective tactical option to combat crime. While the financially rewarding of CHIS has been criticised for costing £22 million between 2011 and 2016 across the United Kingdom (BBC, 2017), data from 2015/2016 (Home Office, 2018) have shown that the annual societal costs for fraud (£4.7 billion), domestic burglary (£4.1 billion) and murder (£1.8 billion) are disproportionate to the cost of running CHIS, who may provide intelligence to prevent such crimes.

Research within covert policing is negligible, none more so than the topic of CHIS. This is because intelligence-related research is confronted by numerous challenges – namely, access to sensitive data, appropriately vetted researchers, an ongoing duty of care, and a failure to recognise that current methods are neither effective nor efficient (Stanier & Nunan, 2018). Intelligence interviews are more informal than evidential interviews undertaken with suspects, victims and witnesses. Though akin to investigative interviews, intelligence interviews are fundamentally an attempt to obtain a narrative of what was witnessed (Billingsley, Nemitz, & Bean, 2001). Previous research has explored ways of maximising the quantity and quality of an account, by exploring the interviewers’ use of rapport (Alison, Alison, Noone, Elntib, & Christiansen, 2013; Collins & Carthy, 2019; Kieckhaefer, Vallano, & Schreiber Compo, 2014; Vallano, Evans, Schreiber Compo, & Kieckhaefer, 2015). There is now a consensus among practitioners and academics that rapport serves as an influential factor in eliciting information from a human source (Abbe & Brandon, 2013; Borum, Gelles, & Kleinman, 2009; Collins, Lincoln, & Frank, 2002; Vallano & Schreiber Compo, 2015).

## Defining rapport

Rapport is considered essential for both Law Enforcement Agencies (LEA) investigations (Caproni, 2008) and intelligence gathering purposes (U.S. Army Field Manual, 2006). This is because it outperforms accusatorial approaches (Evans et al., 2013) as it increases the information gained, enhances cooperation and increases trust (Alison & Alison, 2017). Some practitioners have defined rapport with regards to mutual respect and trust, while others have discussed it in terms of responsiveness to the interviewer (Russano, Narchet, Kleinman, & Meissner, 2014). Additionally, rapport has been noted as the smoothness of the interaction, rather than characterising the individual (Abbe & Brandon, 2013). Such smoothness of the interaction aligns to rapport within a clinical setting, whereby therapists discussed the importance of creating a *therapeutic alliance* (e.g. Bedi, Davis, & Williams, 2005) via the ‘harmonious, sympathetic connection to another’ (Newberry & Stubbs, 1990, p. 14).

So, defining rapport as a working relationship may provide some clarity (Abbe & Brandon, 2013; Vallano et al., 2015). This is because the *working alliance* concerns respect, empathy and a shared understanding of each other’s goals, and lowers an interviewee’s anxiety (Beune, Giebels, & Taylor, 2010, Kelly, Miller, Redlich, & Kleinman, 2013). Further, a *working alliance* is similar in nature to Kleinman’s (2006) concept of operational accord, which goes beyond the broad definitions of rapport, by ensuring that the interviewer and interviewee have shared goals and cooperate. In the context of human intelligence gathering, rapport can be defined as ‘developing and maintaining a working relationship with a human source, by managing their motivations and welfare, whilst ensuring they understand the purpose of the relationship in order to secure reliable intelligence’ (Stanier & Nunan, 2018, p. 232).

## Rapport-based interviewing

Surveys of police officers have commonly discussed rapport as an integral part of interviewing. For example, a questionnaire of 221 police interviewers from the United Kingdom revealed that rapport building was thought to be utilised 87% of the time (Dando, Wilcock, & Milne, 2008). However, police investigators from the United States noted rapport as the fourth most used tactic, and that 32% of investigators always build rapport in their interrogations (Kassin et al., 2007). Redlich, Kelly, and Miller (2014) surveyed U.S. military and federal interrogators regarding their perceived effectiveness and frequency of interrogation techniques. Rapport- and relationship-building techniques were perceived as the most effective strategy, regardless of the intended outcome and context of the interrogation, and, more importantly, rapport- and relationship-building techniques were used most often, especially when compared to confrontational techniques (Redlich et al., 2014).

Goodman-Delahunty and Howes (2016) interviewed 123 experienced intelligence and investigative interviewers about rapport from five Asian-Pacific countries. The rapport-building techniques discussed were primarily represented by liking and reciprocity. Their results supported the generalisability of social influence theory to a range of jurisdictions, as well as reinforcing an earlier international survey that reported that practitioners believe rapport is critical for interviewee cooperation (Goodman‐Delahunty, Martschuk, & Dhami, 2014; Goodman-Delahunty & Sivasubramaniam, 2013). Furthermore, U.S. police officers self-reported that they use, on average, three rapport-building techniques per interview process (Vallano et al., 2015). The most common techniques comprised self-disclosure, sympathy or empathy, and establishing common ground (Vallano et al., 2015).

However, as with the majority of self-reported studies regarding rapport, it was not possible for researchers to observe the recorded interviews to verify what the participants reported (Alison & Alison, 2017). For example, Hall (1997) revealed that police officers reported rapport as important, yet when their recorded interviews were examined, the rapport-building behaviours identified by the police officers were not present. Thus, an objective measure of rapport would provide evidence as to which verbal and nonverbal behaviours actually help establish and maintain rapport (Walsh & Bull, 2012), based on the behaviours that occurred during the interview (Collins & Carthy, 2019). Yet, despite the importance placed on rapport, it is only recently that researchers have begun to explore its actual impact on information gathering in an operational setting (e.g. Alison et al., 2013; Holmberg & Madsen, 2014; Vallano & Compo, 2011).

## Assessing rapport in an operational setting

Limited research has investigated real operational field data to carefully and systematically define the behaviours that underpin rapport. Alison et al. (2013) developed ORBIT (Observing Rapport Based Interpersonal Techniques) from the counselling literature, which is founded on observing interpersonal skills (Tickle-Degnen & Rosenthal, 1990), particularly motivational interviewing (Miller, Moyers, Ernst, & Amrhein, 2008; Miller & Rollnick, 1992) and the interpersonal behaviour circle (Birtchnell, 2014; Freedman, Leary, Ossorio, & Coffey, 1951; Leary, 1957).

Alison et al. (2013) examined 418 real-life terrorist suspect interviews by conducting a structural equation modelling of rapport and its impact on intelligence yield. The ORBIT framework revealed that techniques aligned to motivational interviewing were positively associated with adaptive interpersonal behaviours from the suspect, which resulted in an increased intelligence yield (Alison et al., 2013). Additionally, they reported that minimal maladaptive behaviours from the interviewer directly reduced the intelligence yield. Similar results were reported by Alison et al. (2014), as suspects’ use of counter-interrogation tactics (e.g. no comment interviews, retraction of statements or claiming lack of memory) was reduced when adaptive rapport-based interrogation style (e.g. the use of respect, dignity and integrity) was used.

Similar to the ORBIT framework, a new approach to measuring rapport was established by Collins and Carthy (2019) research, which developed from the Tickle-Degnen and Rosenthal (1990) model of measuring rapport. This was because monitoring the degree of *attention*, *positivity* and *coordination* may provide an insight into the current state of rapport, and whether the interviewee is becoming more or less receptive (Collins & Carthy, 2019). In their study, they analysed 82 suspect interview transcripts regarding sexual offences across three verbal rapport categories against investigative relevant information (see Collins & Carthy, 2019, for a full list of behaviours). The interviewers’ verbal behaviours were classified into one of the three categories of rapport (e.g. *positivity*, *attention* and *coordination*; see later for further discussion), and the sum of each category was calculated. The most frequently used rapport components that were found were *attention* and *coordination*, and both positively correlated with the gathering of investigative relevant information, though *positivity* did not (Collins & Carthy, 2019). The findings provided support for an objective model of measuring rapport, by calculating the frequency of verbal behaviours (which were theoretically and empirically linked to the rapport literature) and their association with information relevant to an investigation.

## Operationalising the Tickle-Degnen and Rosenthal model of rapport

The Tickle-Degnen and Rosenthal (1990) model outlined three components of rapport – namely, *attention*, *positivity* and *coordination*. Rapport is said to develop when all three components are reciprocated during an interaction (Abbe & Brandon, 2014). Although their model of rapport has primarily been discussed within the context of investigative interviewing (Abbe & Brandon, 2014; Collins & Carthy, 2019; Walsh & Bull, 2012), it can also transfer into an intelligence interviewing context. This is because (a) they both aim to obtain reliable and detailed information (information is the raw product of evidence and intelligence; Meissner, Surmon-Böhr, Oleszkiewicz, & Alison, 2017; Russano et al., 2014); and (b) they both concern the interviewing of witnesses, albeit, informants are ‘a special type of witness, but a witness nonetheless’ (Billingsley et al., 2001, p. 7).

*Attention* signifies the degree of involvement and is believed to be present when the parties involved are interested in one another (Holmberg & Madsen, 2014). Thus, the interviewer (source handler) and interviewee (CHIS) begin to direct their focus onto the other, reinforcing a sense of cohesion. In earlier interactions, attentiveness may reinforce the continuation (or not) of the relationship, whereas later attentiveness would signify the level of cohesion (Tickle-Degnen & Rosenthal, 1990). Thus, the level of *attention* should not change over time in order to maintain the developed relationship. Walsh and Bull (2012) demonstrated that establishing rapport alone was not enough to satisfy the interview’s quality and outcomes, as rapport needs to be maintained throughout.

Active listening, not interrupting (Milne & Bull, 1999) and attentiveness (Collins et al., 2002) have been emphasised as beneficial interviewing skills, as such behaviours encourage the interviewee to engage (St-Yves, 2006). The use of back channel responses (i.e. encouragers such as ‘hmm’), paraphrasing and summarising what has previously been said demonstrate active listening and thus *attention* paid to the individual providing their account (Abbe & Brandon, 2013, 2014; Collins & Carthy, 2019; Walsh & Bull, 2012). Once the initial free recall has been provided, exploring and probing the information provided, as well as providing the interviewee with the chance to add anything else, have been discussed as skilful interviewing behaviours (Walsh & Bull, 2012). Throughout the interaction, the source handler should be listening out for and probing information to help identify the CHIS’ motivation. By understanding why a CHIS is willing to engage, this may provide rapport-building opportunities (Cooper, 2011), adapt the approach used (Taylor, 2002) and motivate the CHIS to engage with memory retrieval (Abbe & Brandon, 2013). The source handler and CHIS must engage in some level of *attention* before *positivity* can be established (Abbe & Brandon, 2013).

*Positivity*, the second facet of the model, represents the friendly, respectful and caring nature between all parties involved, which facilitates practical working outcomes (Cuddy, Fiske, & Glick, 2008; Fiske, Cuddy, & Glick, 2007; Tickle-Degnen & Rosenthal, 1990). The use of empathy has been noted to facilitate rapport (Norfolk, Birdi, & Walsh, 2007) and is viewed as a positive behaviour that skilful interviewers utilised (Bull & Cherryman, 1996). Although empathy has not been found to directly influence information gathering (see Oxburgh, Ost, Morris, & Cherryman, 2014), convicted offenders have reported that a humanitarian and empathetic approach fostered their confessions (Holmberg & Christianson, 2002). The use of empathy, together with personalising the interview (e.g. by using preferred names) may be vital to the interview’s overall success (Fisher & Geiselman, 1992).

The disclosure of personal information can also help personalise the interview and has been shown to increase the *positivity* of an interaction (Collins & Miller, 1994), as it demonstrates liking and trust (Abbe & Brandon, 2014). However, the use of self-disclosure by source handlers must be undertaken in a way that reveals sufficient and appropriate information to build rapport (e.g. favourite football team), but does not compromise their own safety by inappropriately revealing overly personal information such as their home address or children’s school (though what is deemed appropriate and inappropriate will be judged differently across CHIS). Where additional information is required to maintain rapport, then source handlers will consider developing appropriate cover stories in order to disclose non-attributable information. Self-disclosure has been found to result in less inaccurate information, protect against misinformation (Vallano & Compo, 2011), and increase the number of agreements reached (Moore, Kurtzberg, Thompson, & Morris, 1999). Vallano and Compo (2011) argued that an informal approach supported by verbal rapport techniques (e.g. self-disclosure) can enhance rapport and memory recall. Such informality suits the source handler and CHIS relationship, as interactions are typically informal, undertaken via the telephone and physical meetings that are not bound by the formality of England and Wales’ Police and Criminal Evidence Act 1984 (e.g. interview under caution).

Establishing common ground also associates with *positivity*, as it encourages the source handler and CHIS to identify overlapping interests. The shared interests can be as meaningful as shared values, or as incidental as a shared birthday (Miller, Downs, & Prentice, 1998). Although is it not persuasive in itself, it can lay the foundations for influence, by prompting those involved to engage and process information more actively (Platow, Mills, & Morrison, 2000). Furthermore, of the three components, *positivity* may be likened to a *working alliance*, especially as friendliness and encouraging comments have been shown to be better predictors of a *working alliance* than *attention* (Duff & Bedi, 2010). While discussions of rapport typically place the most emphasis on *positivity*, *coordination* may be equally, if not more, important for interviewing (Abbe & Brandon, 2014).

As both *attention* and *positivity* grow, the rapport-building process promotes the third component, *coordination* (Holmberg & Madsen, 2014). *Coordination* symbolises the smoothness of interactions, exemplified by a feeling of cooperation and synchrony between the parties involved (Tickle-Degnen & Rosenthal, 1990). Abbe and Brandon (2013) introduced shared understanding into the *coordination* component. By doing so, they argued that a shared understanding between the parties can be pre-existing or established during the interaction. A shared understanding (e.g. agreement) reinforces the common goal or *working alliance* mentality, especially when the purpose of the interaction and developing relationship are discussed (Collins & Carthy, 2019). Such conversations between source handlers and CHIS should encourage the CHIS to provide their account.

Behaviours of *coordination* should directly benefit memory retrieval, particularly when the source handler minimises disruptions, such as appropriately using pauses (Abbe & Brandon, 2013). In line with cooperation, *coordination* requires a balanced conversation, otherwise the interview can become one-sided (Holmberg & Madsen, 2014). However, in an interviewing context, if the interviewee is predominately doing the talking, then the transfer of control has been successful. Therefore, the individual with the information is talking (Collins & Carthy, 2019), and the parties involved are working towards the interview’s aim (e.g. to gather intelligence on a subject of interest or organised crime group).

## The present research

The present research developed the K. Collins and Carthy (2019) verbal rapport framework so that it may be applied to a source handler and CHIS context. Following the recommendations from Abbe and Brandon (2013, 2014), the developed framework investigated rapport behaviours affiliated to Tickle‐Degnen and Rosenthal's (1990) three rapport components; (a) *attention*; (b) *positivity*; and (c) *coordination*. The present research quantified both the rapport behaviours displayed by the source handler and the intelligence yielded from the CHIS, in order to investigate the frequency of these rapport components and their relationship to intelligence yield. It was hypothesised that an increase in *overall rapport* would positively correlate with the amount of intelligence yielded.

## Method

### Materials

The present study expanded on (Nunan, Stanier, Milne, Shawyer, & Walsh, 2020), which explored source handlers’ perceptions of rapport during CHIS interactions, by analysing rapport-building between source handlers and CHIS. Prior to data access and collection, ethical approval was authorised by those who funded the present research (Centre for Research and Evidence on Security Threats) together with the first author’s University. The National Police Chiefs’ Council (NPCC) Intelligence Practice Research Consortium (IPRC) assisted with access to the data. The present research analysed the same data set from (see Nunan et al., 2020), and, therefore, the criteria remained the same. Thus, the purposive sample comprised source handler and CHIS audio recorded telephone interactions (*N*** **=** **105) from one English police force, which involved source handlers’ gathering intelligence (intelligence yield, IY) from an adult CHIS (those who are 18** **years and over). The following telephone interactions were excluded: (a) missed calls; (b) voicemails; (c) interactions that did not concern the collection of intelligence, such as arranging a physical meeting between the source handler and CHIS; or (d) interactions that were merely to arrange a call back (e.g. ‘I can’t talk now, I’ll call you back later’). From 495 interactions across seven source handlers and seven CHIS, a total of 105 interactions were put forward for analysis. The interactions were grouped in order to analyse the verbal rapport behaviours, rather than explore individual performance. The telephone interactions took place in 2018 with a mean call length of 7.03** **min (*SD* = 3.55).

### Procedure

At the time when the telephone interactions took place, the source handlers were unaware that their interactions would be analysed, to ensure that their *normal* verbal behaviours took place. The telephone interactions were approved by the Dedicated Source Unit Controller (source handler supervisor) to ensure that the research team accessed interactions that involved a closed investigation. The first author was required to code all telephone interactions at a secure policing site due to the sensitive nature of the data. To fully comprehend the context of the telephone interaction, the first author listened to the interaction in full before coding during a second listen. It was only possible to analyse verbal rapport as the research team had access to audio recordings of the telephone interactions.

In contrast to the Collins and Carthy (2019) methodology, the present research did not divide the interactions into three equal time segments (i.e. beginning, middle and end). The reasoning was twofold: firstly, the present source handler and CHIS telephone interactions were much shorter in length than a typical investigative interview. Secondly, dividing an interaction into three equal segments is unlikely to truly represent the ‘beginning’, ‘middle’ and ‘end’ of an interaction, if the beginning is to represent the introductions to the interview, the middle as the account phase, and the end as the closure phase. Therefore, verbal rapport was analysed across the whole interaction.

### Verbal rapport coding

The present framework of verbal rapport developed Collins and Carthy (2019) measures of interpersonal rapport (see Table 1). The framework of verbal rapport used an objective measure of rapport, by coding the frequency of each verbal behaviour, rather than using a subjective rating scale (e.g. a Likert scale of 1–5) of rapport behaviours or the interaction as a whole. Each word or phrase from the source handler was only coded as one of the three rapport components (e.g. *attention*, *positivity* or *coordination*) from the developed framework (see Table 1) and could not be coded as multiple components. The sum for all three components of rapport was calculated.

**Table 1.** A framework of verbal rapport for source handler and CHIS interactions.

Component	Rapport indicator	Definition	Source
Attention	Back channel responses	Back channel responses act as facilitators/encourages, e.g. ‘uh huh’ or ‘hmm’, this does not include qualitative feedback such as ‘perfect’ or ‘good’ as these can be viewed as leading and therefore negative.	Abbe & Brandon, 2013, 2014; Collins & Carthy, 2019
	Paraphrasing	Repeating back what the CHIS said, which demonstrates the source handler has clearly attempted to process what the CHIS is saying.	Abbe & Brandon, 2014; Alison et al., 2013; Collins & Carthy, 2019.
	Identifying emotions	The source handler attends to the CHIS’ emotions, e.g. ‘you sound upset’.	Alison et al., 2013; Collins & Carthy, 2019
	Explores and probes information	Goes beyond just accepting information but searches for further detail, identifying the provenance of the information provided, funnel from open to closed questioning.	Alison et al., 2013; Walsh & Bull, 2012
	Intermittent summarising	Provides regular and accurate summarising of the CHIS’ account.	Abbe & Brandon, 2014; Alison et al., 2013; Walsh & Bull, 2012
	Provides final summary of interaction	Final summary that accurately resumes key issues discussed and captures key proses from the CHIS.	Walsh & Bull, 2012
	Asks if the CHIS wishes to add or alter anything	Provides opportunity for the CHIS to make any amendments or additions to their account.	Walsh & Bull, 2012
	Explores motivation	Tries to find, with understanding, why the CHIS is willing to share their information and also use the CHIS’ motivation for the conversation. Source handler may use the motivation as a hook for cooperation.	Abbe & Brandon, 2013; Taylor, 2002
Positivity	Use of CHIS’ preferred name	‘Where did you buy the computer James?’	Abbe & Brandon, 2014; Collins & Carthy, 2019; Collins et al., 2002; Kieckhaefer et al., 2014; Vallano & Compo, 2011; Walsh & Bull, 2012
	Empathy	A sensitive approach demonstrated by empathic responses, e.g. ‘I can understand why you might feel nervous’.	Abbe & Brandon, 2014; Alison et al., 2013; Collins & Carthy, 2019; Beune et al., 2010; Holmberg & Madsen, 2014; Oxburgh et al., 2014; Walsh & Bull, 2012
	Self-disclosure	When you feel you have learned something about the source handler that you didn't know before, e.g. ‘I have children too’.	Abbe & Brandon, 2014; Kieckhaefer et al., 2014; Nash et al., 2016; Vallano & Compo, 2011; Vallano et al., 2015
	Common ground by getting to know the CHIS	The use of questions around the CHIS’ lifestyle, hobbies, family etc. to display a genuine interest towards the CHIS	Abbe & Brandon, 2014; Kieckhaefer et al., 2014; Holmberg & Madsen, 2014; Nash et al., 2016; Vallano et al., 2015
	Equality signs/ Friendliness	Matches CHIS’ style and does not belittle or talk condescendingly to or ‘above’ the CHIS. Is polite, friendly, respectful and courteous, e.g. ‘thank you’; ‘how are you feeling today?’.	Abbe & Brandon, 2013; Alison et al., 2013; Beune et al., 2010; Collins & Carthy, 2019; Collins et al., 2002; Holmberg & Madsen, 2014; Vallano & Compo, 2011; Vallano et al., 2015; Walsh & Bull, 2012
	Humour	The CHIS must find the use of humour as a positive, e.g. ‘thanks for telling me your age, I know you said your date of birth, but I couldn’t work it out as my maths isn’t all that great (laughs)’	Alison et al., 2013; Collins & Carthy, 2019.
	Reassurance	The source handler provides encouragement and places the CHIS at ease e.g. ‘we will get this sorted’; ‘keep at what you’re doing’.	Collins & Carthy, 2019
Coordination	Agreement	Working towards a common goal or working alliance e.g. ‘yeah that is what I meant’.	Abbe & Brandon, 2013; Collins & Carthy, 2019
	Encourages CHIS to give account	Evidence of explicitly asking the CHIS for their account and allowing the CHIS to give it without any inappropriate interruptions.	Alison et al., 2013; Walsh & Bull, 2012
	Appropriate use of pauses	Source handler uses pauses to facilitate talking, which are not awkwardly placed.	None
	Process, procedure and what happens next	Explains future agenda and processes, any necessary regulatory requirements such as ‘don’t tell anyone about this conversation’, maintain security and welfare, when to next contact, and future taskings.	Abbe & Brandon, 2013; Collins & Carthy, 2019; Nash et al., 2016; Walsh & Bull, 2012

Note: Academic sources were collated with the assistance of Gabbert, Hope, Luther, Ng, Wright, and Oxburgh’s (under review) rapport and disclosure searchable systematic map. CHIS = Covert Human Intelligence Source. For the purposes of this research, the term CHIS has replaced the term interviewee.

The first and second authors utilised the framework of verbal rapport (see Table 1) to code the audio recorded telephone interactions. In order to ensure the coding scheme was viable, the first and second authors coded one telephone interaction together as a training exercise. The second author independently coded a random sample of 13 of the source handler and CHIS interactions. The interrater reliability was calculated using Cohen’s kappa (Cohen, 1960), which revealed a high agreement between the two independent coders, *k* = .77, 95% confidence interval (CI) [.71, .83], *p*** **<** **.001.

### Intelligence yield

The information provided by the CHIS in the present research was coded if it held relevance to criminal intelligence. Intelligence yield (IY) comprised five detail types: (a) *surrounding* details included information about the setting (e.g. locations); (b) *object* details concerned items that were discussed (e.g. a phone, drugs, money); (c) *person* details consisted of information relating to people (e.g. names, person descriptions); (d) *action* details comprised information about activities (e.g. criminal offences, driving); and (e) *temporal* details related to the time (e.g. dates, days, years; see similar coding systems: Hope, Mullis, & Gabbert, 2013; Milne & Bull, 2002; Wessel, Zandstra, Hengeveld, & Moulds, 2015). For example, ‘around 9** **pm (one temporal IY) she (one person IY) was driving (one action IY) a car (one object IY) and dealing (one action IY) drugs (one object IY) in London (one surrounding IY)’. This coding scheme was used to quantify the total IY gathered by the source handler from the CHIS per interaction.

## Results

Across the sample (*N*** **=** **105) of audio recorded telephone interactions between source handlers and CHIS, the means for (a) *overall rapport*; (b) the three components of rapport (i.e. *attention*, *positivity* and *coordination*); (c) overall IY; and (d) five detail types of IY (surrounding, object, person, action and temporal) were explored. Pearson’s correlation coefficients were performed to explore the relationship between *overall rapport*, *attention*, *positivity* and *coordination* with IY. The effect sizes for Pearson’s correlation coefficients were sourced from Cohen (1988), whereby .10 is a small effect, .30 is a medium effect and .50 a large effect. To display the practical importance of the results, the Pearson’s correlation coefficients (*r*) were squared to establish the coefficient of determination (*R*^2^). The coefficient of determination represents the percentage of the observed variation that can be explained by one factor (i.e. *overall rapport*, *attention*, *positivity* or *coordination*) with another factor (i.e. IY) with regards to a linear model (reported in Table 2).

**Table 2.** Coefficients of determinations (*R*^2^) for rapport components against overall intelligence yield and detail type.

Rapport component	Intelligence yield					
	Surrounding	Object	Person	Action	Temporal	Overall
Attention	.41***	.59***	.58***	.65***	.36***	.69***
Positivity	.03	.01	.03	.05*	.00	.04
Coordination	.01	.03	.03	.06*	.05*	.05*
Overall rapport	.29***	.38***	.40***	.48***	.24***	.48***

Note: *N*** **=** **105.

*p** **<** **.050. **p** **<** **.010. ***p** **<** **.001.

### Rapport and intelligence yield

Across the sample, the mean *overall rapport* utilised per interaction was 47.10 (*SD* = 21.75). The *attention* (*M*** **=** **24.77, *SD* = 15.26) component of rapport was the most frequently used, followed by *positivity* (*M*** **=** **12.21, *SD* = 6.53) and then *coordination* (*M*** **=** **10.12, *SD* = 5.23). On average, 87.26 (*SD* = 61.63) IY was gathered per interaction, with the five detail types displayed in Table 3.

**Table 3.** Means and standard deviations for intelligence yield across the telephone interactions.

IY	*M*	*SD*
Surrounding IY	11.74	12.74
Object IY	14.48	12.95
Person IY	26.89	21.87
Action IY	25.56	20.37
Temporal IY	06.11	04.92
Overall IY	87.26	61.62

Note: *N*** **=** **105. IY = intelligence yield.

### The relationship between rapport and intelligence yield

Pearson’s correlation coefficients were undertaken to explore the relationship between the three components of rapport and *overall rapport* with IY.^1^ *Overall rapport* was significantly correlated with overall IY, *r*** **=** **.69, *p*** **<** **.001. When *overall rapport* was broken down into its three components, *attention*, *r*** **=** **.83, *p*** **<** **.001, and *coordination*, *r*** **=** **.21, *p*** **=** **.028, were both significantly correlated with the overall IY gathered (though a high level of variability between *coordination* and IY was revealed). However, there was a non-significant correlation with *positivity* and overall IY, *r*** **=** **.19, *p*** **=** **.051.

To investigate the correlations further, the relationship between the five detail types of IY with *overall rapport* and its three components (*attention*, *positivity* and *coordination*) were also explored (see Table 2 for an *R*^2^ overview). *Overall rapport* was significantly correlated with all five detail types – namely, surrounding IY, *r*** **=** **.54, *p*** **<** **.001; object IY, *r*** **=** **.62, *p*** **<** **.001; person IY, *r*** **=** **.63, *p*** **<** **.001; action IY, *r*** **=** **.69, *p*** **<** **.001; and temporal IY, *r*** **=** **.49, *p*** **<** **.001. *Attention* also significantly correlated with all five detail types: surrounding IY, *r*** **=** **.64, *p*** **<** **.001; object IY, *r*** **=** **.77, *p*** **<** **.001; person IY, *r*** **=** **.76, *p*** **<** **.001; action IY, *r*** **=** **.81, *p*** **<** **.001; and temporal IY, *r*** **=** **.60, *p*** **<** **.001. *Positivity* significantly correlated with action IY, *r*** **=** **.23, *p*** **<** **.050, but not surrounding IY, *r*** **=** **.18; *p*** **=** **.060; object IY, *r*** **=** **.12, *p*** **=** **.221; person IY, *r*** **=** **.17, *p*** **=** **.075; or temporal IY, *r*** **=** **.06, *p*** **=** **.578. *Coordination* significantly correlated with action IY, *r*** **=** **.24, *p*** **<** **.050 and temporal IY, *r*** **=** **.23, *p*** **<** **.050, but not with surrounding IY, *r*** **=** **.12, *p*** **=** **.223; object IY, *r*** **=** **.184, *p*** **=** **.06; and person IY, *r*** **=** **.18, *p*** **=** **.063.

## Discussion

The present research developed the Collins and Carthy (2019) rapport framework and applied it to an intelligence gathering context. Thus, the relationship between rapport and the gathering of intelligence (i.e. intelligence yield, IY) was explored in real-world audio recorded telephone interactions between source handlers and CHIS. To meet the research aims, the relationship and observed variation between *overall rapport* and its three components (i.e. *attention*, *positivity* and *coordination*; see Tickle-Degnen & Rosenthal, 1990) with IY were explored. The research findings provided further support for the application of a systematic framework to measure verbal rapport, utilised by ‘the coding of behaviours that have been theoretically and empirically linked to rapport’ (Collins & Carthy, 2019, p. 27).

*Overall rapport* was significantly correlated with IY and, as an independent factor, explained 48% of the variance within IY. While this finding supports that an increase in *overall rapport* would positively correlate with the amount of intelligence yielded, ultimately it may be argued that the hypothesis is only partially supported. That is, the explained variability in the data set does not account for 52% of the observed variation. Thus, as a reliable model of future forecast, *overall rapport* may not accurately model the data (see, Ozer, 1985, for a more complete report of interpreting the coefficient of determination). While rapport is considered as an influential factor in the elicitation of information from a human source (Abbe & Brandon, 2013; Borum et al., 2009; Vallano & Schreiber Compo, 2015), especially as interviews of greater quality have been positively associated with highly rated rapport behaviours (Walsh & Bull, 2012), it does not appear to be the only factor at play. Understandably so, as within real-world source handler and CHIS interactions (and interviews more broadly), numerous factors may act as a communication barrier or encourager (e.g. elicitation techniques, interviewees’ motivation to engage, memory, policy and procedures). Nonetheless, this finding has provided additional evidence to the existing rapport literature, further highlighting a positive relationship between an interviewer’s behaviour (i.e. rapport) and the elicitation of intelligence (see also Alison et al., 2013; Collins & Carthy, 2019). Frequency monitoring of rapport and its three components can provide an insight into the current state of rapport in an interaction (Collins & Carthy, 2019). Perhaps, more importantly so, is the exploration of the relationship between each component of rapport with the production of IY.

*Attention* was the most frequently used component of rapport, followed by *positivity*, and then *coordination*. A core objective for a source handler is to maintain a working relationship with their CHIS. As attentiveness is considered an important factor to the continuation (or not) of the relationship (Tickle-Degnen & Rosenthal, 1990), this may explain why source handlers utilised this component of rapport the most. The level of *attention* should not change over time as rapport needs to be maintained throughout, in order to satisfy the interview’s quality and outcomes (Walsh & Bull, 2012). Attentive behaviours such as active listening (Milne & Bull, 1999) and probing the information elicited (Walsh & Bull, 2012) may notify the source handler that the communicative approach they are using is suitable to the CHIS in question (Taylor, 2002). Consequently, the appropriate deployment of attentive behaviours should motivate the CHIS to engage with memory retrieval (Abbe & Brandon, 2013), thus benefiting the collection of intelligence. The present research found that *attention* significantly correlated to IY, and explained 69% of the variance of the data, providing support for the positive impact that attentive behaviours of verbal rapport have on the gathering of intelligence.

In contrast to K. Collins and Carthy (2019), the present research revealed that *positivity* was used more frequently than *coordination*. This may be explained by the differences in formality and process between the Collins and Carthy (2019) sample of formal investigative interviews with suspects of sexual offences and the present sample of informal telephone interactions between source handlers and CHIS. As such, the behaviours associated with the *positivity* component of rapport (e.g. humour, empathy and common ground) may be more appropriate and therefore more likely to be used in an informal setting. Additionally, behaviours associated with *coordination* *–* for example, discussing and ensuring the understanding of the process and procedures – are more likely to take place in suspect interviews, in accordance with England and Wales’ Police and Criminal Evidence Act 1984 (see Collins & Carthy, 2019).

Discussions of rapport typically place the most emphasis on *positivity*; however, the present research reported that *positivity* was non-significantly correlated to IY, only explaining 4% of the variance within intelligence yielded. Collins and Carthy (2019) also reported a similar finding, though posited that perhaps the negative attitude towards sex offenders may have explained their finding. However, the present sample consisted of cooperative CHIS in productive relationships with their source handlers, yet still no positive correlation between *positivity* and IY was reported. As source handlers and CHIS in the present research had already established a relationship prior to the interactions analysed (compared to investigative interviewers who typically meet the interviewee for the first time and, with suspects, often immediately after an arrest), the increased familiarity may have resulted in a reduced impact of *positivity*, as it may not have been considered to be as important as *coordination* or *attention* (Abbe & Brandon, 2014; Tickle-Degnen & Rosenthal, 1990).

While *positivity* is commonly discussed with regards to rapport, *coordination* may be more important for interviewing (Abbe & Brandon, 2014). The operationalisation of *coordination* should differ between a source handler and CHIS compared to an interviewer and suspect due to the type of relationship that exists (Collins & Carthy, 2019). Source handlers aim to achieve a *working alliance* with their CHIS, which is an ongoing process, whereas the same level of cooperation is less likely to exist between a suspect and interviewer who may only meet on one or two occasions. Surprisingly *coordination* was found to be the least frequently used component of rapport in the present research, though it was significantly correlated to IY. However, when exploring the variability within the data, *coordination* could only explain 5% of the variance for intelligence yield.

*Coordination* behaviours should directly benefit information gathering, particularly when the source handler appropriately uses pauses (Abbe & Brandon, 2013) and encourages an account (Walsh & Bull, 2012). Furthermore, it is plausible that when the source handler explains the process, procedure and future expectations, as well as developing a shared understanding with the CHIS (e.g. agreement on when next to physically meet up, to be contacted by the telephone, or to receive financial reward payments), communication increases (Abbe & Brandon, 2013; Collins & Carthy, 2019; Nash, Nash, Morris, & Smith, 2016; Walsh & Bull, 2012). However, as source handlers rarely used pauses to facilitate communication and on occasions interrupted their CHIS, this may explain why the *coordination* component of rapport was the least frequently utilised.

At present, nationally delivered source handler training in England and Wales includes little mention of rapport-building techniques. The rapport framework in the present research could be utilised in a training environment to highlight verbal behaviours associated with the three components of rapport. While the frequency of the three components of rapport differed from that in the Collins and Carthy (2019) study, the finding that both *attention* and *coordination* (though a high level of variability was revealed for *coordination*) were significantly related to the amount of information gathered was consistent. In terms of eliciting information, it appears that placing an emphasis on *attention* and *coordination* verbal behaviours of rapport is pragmatic. That said, *positivity* should not be disregarded, as these behaviours may serve a different purpose within interviewing, such as empathy, respect and reassurance to the CHIS. *Positivity* in a law enforcement interaction is unlikely to have the same impact as it would in a therapeutic interaction, as the aims of the two interaction types differ (Abbe & Brandon, 2013).

The present research advocates for the utilisation of the coefficients of determinations (*R*^2^) when examining rapport. This is because the coefficients of determinations go beyond just accepting significant correlations at face value, but rather explore how the percentage of observed variation that can be explained by one factor (i.e. intelligence yield) with another factor (i.e. *overall rapport*, *attention*, *positivity* or *coordination*). This encourages the research findings to be discussed in respect of their practical importance (e.g. the determining predictive power of rapport and its three components). As such, while *coordination* was reported as significantly correlated to intelligence yield, it may only explain 5% of the variance within the intelligence yielded. Therefore, a high level of variability (e.g. 95%) between *coordination* and intelligence yielded was revealed. Although *coordination* could only explain a small percentage of the variability, its statistical significance may suggest it plays a small role in gaining intelligence.

It is important to note the limitations of the present research. As a consequence of working with a sensitive data set reliant on the police providing access, the present sample originates from one police force area. It was necessary to use a purposive sample to analyse interactions that met the inclusion and exclusion criteria. While it is acknowledged that the sample is not random, the present research accessed a unique sample, which was constrained by the research aims and participating organisations, meaning that convenience and purposive sampling methods are common amongst applied research. While the findings may not reflect the general verbal rapport practices of source handlers across England and Wales, the source handlers in the present sample were trained and accredited through the same national course as those employed elsewhere in this role. Additionally, the generalisability may also be limited as a result of grouping the interactions, as the findings may not generalise to the individual level (Klein & Kozlowski, 2000). Future research may try to gather data from a broader range of source handlers, by analysing telephone interactions from numerous police force areas in order to compare and contrast practices. Finally, while the present research focused on the verbal rapport behaviours of the source handler concerning intelligence yield, it is acknowledged that rapport is a dyadic relationship. Therefore, future research may wish to include the CHIS’s behaviour, as it would enable the researchers to explore reciprocal aspects of the interaction.

In conclusion, the present research has developed a systemic framework of verbal rapport, which was, for the first time, successfully applied to real-world audio recorded telephone interactions between source handlers and CHIS. The results provided additional evidence that rapport is an influential factor to intelligence elicitation. In particular, the findings indicated that the frequency of the rapport components, as well as the verbal rapport behaviours associated with *attention* and *coordination*, had the most impact on the elicitation of intelligence.

The present research holds a number of implications for source handler training, policy and practice. The significance of the rapport and its three components should be incorporated into source handler training, especially as the present framework of rapport could be used to assess training sessions and monitor real-world interactions. Moreover, if source handlers were to place an emphasis on both *attention* and *coordination*, this may benefit the elicitation of intelligence. The implementation of an evidence-based approach to rapport and information gathering shall advance the practices of source handlers and interviewers more broadly.

## Footnotes

1 The three components of rapport were positively correlated: attention and positivity, *r* = .35, *p* < .010; attention and coordination *r* = .38, *p* < .010; and coordination and positivity *r* = .60, *p* < .010.
